# Multifunctional THz Graphene Antenna with 360^∘^ Continuous *ϕ*-Steering and *θ*-Control of Beam

**DOI:** 10.3390/s23156900

**Published:** 2023-08-03

**Authors:** Victor Dmitriev, Rodrigo M. S. de Oliveira, Rodrigo R. Paiva, Nilton R. N. M. Rodrigues

**Affiliations:** Graduate Program in Electrical Engineering (PPGEE), Institute of Technology (ITEC), Federal University of Pará (UFPA), Rua Augusto Corrêa, 01, Belém 66075-110, PA, Brazil; rods.paiva26@gmail.com (R.R.P.); niltonrodolfo@ufpa.br (N.R.N.M.R.)

**Keywords:** THz communications, dipole graphene antenna, multifunctional antenna, reconfigurable radiation pattern, beam steering

## Abstract

A novel graphene antenna composed of a graphene dipole and four auxiliary graphene sheets oriented at $90^{\circ }$ to each other is proposed and analyzed. The sheets play the role of reflectors. A detailed group-theoretical analysis of symmetry properties of the discussed antennas has been completed. Through electric field control of the chemical potentials of the graphene elements, the antenna can provide a quasi-omnidirectional diagram, a one- or two-directional beam regime, dynamic control of the beam width and, due to the vertical orientation of the dipole with respect to the base substrate, a $360^{\circ }$ beam steering in the azimuth plane. An additional graphene layer on the base permits control of the radiation pattern in the θ-direction. Radiation patterns in different working states of the antenna are considered using symmetry arguments. We discuss the antenna parameters such as input reflection coefficient, total efficiency, front-to-back ratio, and gain. An equivalent circuit of the antenna is suggested. The proposed antenna operates at frequencies between 1.75 THz and 2.03 THz. Depending on the active regime defined by the chemical potentials set on the antenna graphene elements, the maximum gain varies from 0.86 to 1.63.

## 1. Introduction

Terahertz (THz) communication systems, operating in the frequency range of 0.1 THz to 10 THz, have emerged as a promising solution to address the ever-increasing demand for high-speed wireless communication. This frequency range, known as the THz gap, lies between the traditional microwave and infrared bands, offering unique opportunities for a wide range of applications, including sensing [1], imaging [2], spectroscopy [3], and high-speed data transmission antennas [4,5,6,7].

Graphene is a material formed by a single layer of carbon atoms arranged in a honeycomb lattice [8]. Graphene possesses specific electronic and electrical properties, in particular, voltage-controlled chemical potential and, consequently, the possibility for dynamic control of its complex electric conductivity [9,10]. Application of graphene has led to significant technical advances in several fields [9,11,12,13,14], including design of nanoantennas for various applications [4], such as intra- and inter-chip communications. In the THz region, graphene supports surface plasmon polaritons (SPPs) [9], providing the small dimensions of circuit components. During the last decade, different types of graphene antennas have been suggested in the literature, starting from a simple dipole one to more complex structures presenting combinations of graphene with metal elements or with dielectric resonators. Many of them have been projected by analogy with microwave [15] and optical antennas [16], such as bow-tie, loop, Yagi–Uda, spiral, and log-periodic ones.

A review of microwave beam-switchable antennas is presented in [17]. The methods of beam switching in the microwave frequency region are based on the use of phased array antennas, liquid metal antennas, antennas based on active frequency selective surfaces, and electronically controlled quasi-Yagi array antennas. Notice that some of these methods can also be applied in the THz region using graphene as a tunable material.

Today, the number of the papers devoted to graphene antenna technology is in the hundreds. Detailed descriptions of many graphene and graphene-based antennas are given, as in the review papers [4,18]. In [18], the author discusses graphene antenna theory and experiments at that early stage, such as the graphene patch, plasmonic resonant antennas, and reflectarrays. A detailed discussion of graphene antenna technology is presented in a recently published review paper [4]. There, one can find a wide spectrum of issues, starting from the fundamental principles of the graphene antenna theory to the description of the technology in fabrication of graphene-–metal nanoantennas. Many graphene antenna structures are presented the reference paper. A considerable part of the paper is devoted to the methods for radiation beam scanning and beam reconfigurability. Hence, there is no need to discuss the published literature in detail. Therefore, we decided to restrict our literature review to recent publications that did not appear in the existing review papers and to the works where the suggested antennas possess some functionalities similar to our proposal.

In paper [19], a monopole source antenna is surrounded by six hexagonal active frequency selective surfaces. These surfaces permit stepwise $0^{\circ }$, $\pm 60^{\circ }$, $\pm 120^{\circ }$, and $180^{\circ }$-switching of the beam in two different frequency bands of 0.96 THz and 1.21 THz. However, the proposed antenna is very complex in its biasing graphene scheme and has a complex geometry and large dimensions defined by the hexagonal screen. The THz antennas proposed in [20,21], based on the Yagi–Uda antenna concept, consist of two graphene-based dipoles and graphene-based parasitic elements. These elements can act as directors or reflectors by controlling their surface conductivity. The antennas can direct the main lobe into four orthogonal θ-directions: $0^{\circ }$, $\pm 90^{\circ }$, or $180^{\circ }$.

The antenna in [22] consists of a graphene dipole with two coplanar graphene parasites placed on the SiO_2_ substrate. Working in the frequency range of 1.94–2.13 THz, the antenna has four radiation operation states that can be selected by setting specific chemical potentials using the external electric field on the graphene elements. In state 1, a dipole-like radiation diagram is produced. States 2 and 3 are characterized by specific directional radiation patterns. By selecting the operation state 4, the antenna is switched off. In [5], a rectangular graphene loop antenna placed on a dielectric substrate for the THz band is proposed and analyzed. Symmetry properties of the antenna in terms of currents and the radiated fields are investigated. The maximum total efficiency of the proposed antenna is about 57%, and its fractional bandwidth is 96%, with the central frequency 1.74 THz. This bandwidth is approximately twice that of the graphene dipole antenna.

Recently, an antenna containing an active radiating graphene patch and a non-radiating graphene ring was analyzed in [23], where the radiation pattern can be steered by controlling gate voltages over the ring. A reconfigurable graphene leaky-wave antenna with electronic beam scanning for a THz communications system is proposed in [24]. It consists of graphene strips printed on a silicon oxide substrate and fed by a planar H-plane horn antenna. The tunable graphene conductivity using DC-bias is used to control the radiated beam direction. By selecting the periodicity of the biased/unbiased graphene strips of the antenna, the beam direction, scanning range, and gain can be controlled. The radiated beam is electronically scanned from −$68^{\circ }$ to $26^{\circ }$ at the frequency 2 THz. A THz beam steering microstrip patch antenna is demonstrated in [25]. The metallic radiating patch is surrounded by six parasitic graphene ribbons located at the lateral, upper, and lower sides of the radiating patch. The graphene ribbons are employed to change the beam orientation in the θ-constant plane. The chemical potentials for graphene elements are switched between 0 and 1 eV to produce beam steering. Two lateral ribbons act as director elements, whereas four upper and lower parasitic elements take on the reflector role, resulting in a beam deflected into the opposite direction. As a result, an overall $120^{\circ }$ beam steering at the frequency of 1.47 THz is obtained.

In [26], opto-electronic simulation is carried out, and the emission intensity spectrum is determined in a graphene-based THz bow-tie dipole antenna on a substrate with photonic band gap structure. The directivity of the proposed antenna with photonic V is found to be 13.7 dBi, which is a 10 dB improvement over the conventional design, and an efficiency of 95% is achieved. A Ti–Au dipole antenna on a GaAs substrate is designed in [27] for THz emission. A spectral width of 120 GHz is obtained from the emission spectrum. In order to compare the spectral characteristics, a graphene dipole antenna is designed on the same substrate. It is observed that the graphene dipole yields a narrower spectral width of 70 GHz due to its high Q-factor.

A graphene multiple-input multiple-output (MIMO) microstrip patch antenna in [28] contains graphene E-shaped elements placed between the graphene radiating patches. It provides a higher isolation between the unit cells. At the frequency 1.9 THz, the antenna produces the radiated beam, allowing it to steer in different directions within the angle interval $\pm 60^{\circ }$. The reconfigurable process is carried out by changing the chemical potential of the antenna elements.

In [29], a sub-THz emitter based on a large-bandwidth silicon-plasmonic graphene photodetector integrated with a broadband rounded bow-tie THz antenna was fabricated. The sub-THz emitter is experimentally demonstrated to emit sub-THz waves with a radiation spectrum from 50 to 300 GHz. A maximum sub-THz emission power of 5.4 nW is obtained at 145 GHz with 3 mW input light power. The emitter can be fabricated by a CMOS-compatible process.

In this paper, we propose a very simple antenna consisting of a graphene dipole, two coplanar reflectors, and two orthogonal reflectors also made of graphene. The main peculiarity of our antenna is its multifunctionality. We will show that it can work in the omnidirectional state and in five other discrete states that differ in the form of the diagram pattern. The control of these states is fulfilled by a simple change to the chemical potential of the graphene elements. In addition to these states, due to vertical orientation of the dipole with respect to the base substrate, there is a possibility of continuous $360^{\circ }$ ϕ-steering of the beam and also θ-control of it. The analysis of the published literature presented above demonstrates that only a stepwise $360^{\circ }$ control of the THz beam in graphene antennas has been suggested up to now. The reported continuous beam steering is restricted by a relatively small ϕ-angle around $\pm 60^{\circ }$. Our proposed antenna is very simple; it is small and has a high level of reconfigurability that has not been achieved until now.

The paper is organized as follows. The antenna description is presented in Section 2. Graphene parameters used in calculations are discussed in Section 3. Section 4 is devoted to symmetry analysis of the proposed antenna. Section 5 provides the qualitative analysis of currents and fields in the antenna. In Section 6, we present the design of the dipole antenna. The design of the antenna with four reflectors is discussed in Section 7. The results of the numerical simulations are detailed in Section 8. A discussion and conclusions end the paper with Section 9 and Section 10, respectively.

## 2. Antenna Description

**Figure 1:**
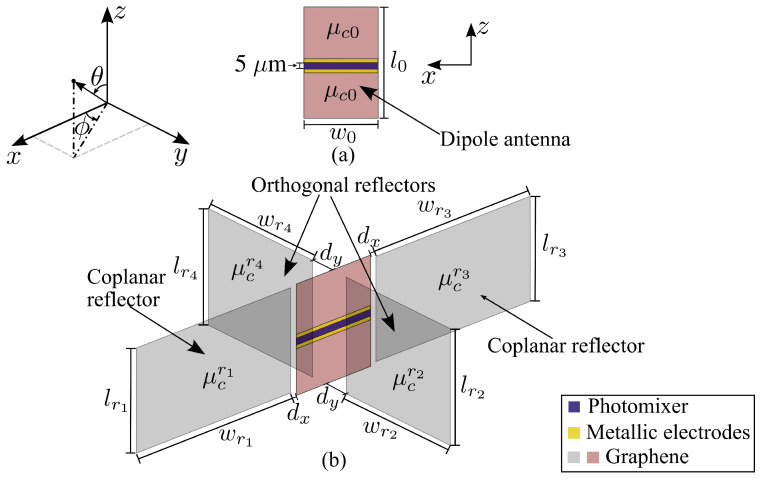
The proposed device: (**a**) dipole antenna and (**b**) dipole and four reflectors.

Geometry of the proposed antenna in free space is presented in Figure 1.

The device is formed by an active graphene dipole antenna (measuring $l_{0}\times w_{0}$), which is fed by a photomixer [30] placed in the gap between the dipole arms. The photomixer and the metallic electrodes are 5μm and 0.5μm in width, respectively. Two orthogonal reflectors with respect to the plane of the dipole with dimensions $l_{r_{2}}$, $w_{r_{2}}$, $l_{r_{4}}$, and $w_{r_{4}}$, and two coplanar graphene reflectors, measuring $l_{r_{1}}\times w_{r_{1}}$ and $l_{r_{3}}\times w_{r_{3}}$, are also a part of the device. The distance between the dipole antenna and each coplanar reflector is $d_{x}$, while $d_{y}$ is the separation between the dipole and each orthogonal reflector. The dipole arms and orthogonal reflectors are made of three-layer graphene sheets, while each coplanar reflector consists of a single-layer sheet.

Thus, the discussed system consists of a common graphene dipole antenna shown in Figure 1a and four graphene reflectors oriented at $90^{\circ }$ to each other (see Figure 1b). The reflectors that provide beam shaping present equal rectangular graphene sheets placed symmetrically with respect to the active dipole element. The geometric configuration among the graphene dipole and reflectors and the vertical orientation of the graphene dipole antenna with respect to the base substrate, as discussed later, provide the possibility of $360^{\circ }$ beam steering. A possible implementation of chemical potential tuning is discussed in Section 9.

## 3. Graphene Parameters

The chemical potential of the dipole antenna is $\mu_{c0}$, while chemical potentials of orthogonal reflectors are $\mu_{c}^{r_{2}}$ and $\mu_{c}^{r_{4}}$. Coplanar reflector chemical potentials are $\mu_{c}^{r_{1}}$ and $\mu_{c}^{r_{3}}$ (see Figure 1). In the THz band, the complex conductivity of a single layer of graphene is properly described by its intraband conductivity contribution [5,9], given by

(1) $$\sigma_{g}=\frac{q_{e}^{2}k_{B}T}{\pi \hbar^{2}\left(j\omega +2\Gamma_{g}\right)}\left[\frac{\mu_{c}}{k_{B}T}+2\ln\left(1+e^{-\mu_{c}/k_{B}T}\right)\right],$$

where $q_{e}$ is the electron charge, $k_{B}$ and *ℏ* are the Boltzmann’s and the reduced Planck’s constants, *T* is temperature, $j=\sqrt{-1}$, ω is angular frequency, $\mu_{c}$ is chemical potential, and $\Gamma_{g}={\left(2\tau \right)}^{-1}$ is the scattering rate (τ is relaxation time). The conductivity of a graphene sheet with N≤5 layers is $\sigma_{N}=N\sigma_{g}$, as detailed in [9,31,32].

## 4. Symmetry Analysis

### 4.1. The Full 3D Symmetry of the Antenna

The physical symmetry of the discussed antenna is defined by the geometric symmetry of the structure and by the symmetry of chemical potentials of the reflectors. The resulting symmetry defines the distribution of currents in the graphene elements and, consequently, the distribution of the electromagnetic field around the antenna. Therefore, symmetry of the radiation pattern (RP) of the antenna, which is the main subject of our work, depends on the resulting symmetry of the antenna.

Group theory greatly simplifies the description, calculations, and analysis of the systems with high symmetry. The highest symmetry of the free-standing antenna in Figure 1b is described by the three-dimensional (3D) point group $D_{2h}$ (in Schoenflies notation [33]). This group consists of the following elements: *e* is the unit element; $C_{2z}$ is a twofold principal symmetry axis *z*; $C_{2}^{\prime }$ and $C_{2}^{\prime \prime }$ are two twofold symmetry axes orthogonal to the principal axis; *i* is inversion through a center of symmetry; $\sigma_{x}$ is the vertical plane y=0 aligned with the principal symmetry axis; $\sigma_{y}$ is the vertical plane x=0 aligned with the principal symmetry axis; and $\sigma_{z}$ is a horizontal plane intersecting the principal symmetry axis.

The group $D_{2h}$ can be presented as the direct product $D_{2h}=C_{2v}⊗C_{s}$ of two lower groups. The 2D group $C_{2v}$ describes symmetry in the plane x0y, and the group $C_{s}$, consisting of the unit element *e* and the plane of symmetry $\sigma_{z}$, describes symmetry in the third coordinate *z*. The plane of symmetry $\sigma_{z}$ allows one to consider only the upper (z>0) or lower (z<0) half-space. In Section 8.5, we demonstrate the effect of symmetry reduction, deleting the plane $\sigma_{z}$. In the following, we restrict ourselves to the group $C_{2v}$. This group contains the elements *e*, $C_{2}$, $\sigma_{x}$, and $\sigma_{y}$.

### 4.2. Effect of Dielectric Substrates on Antenna Symmetry

**Figure 2:**
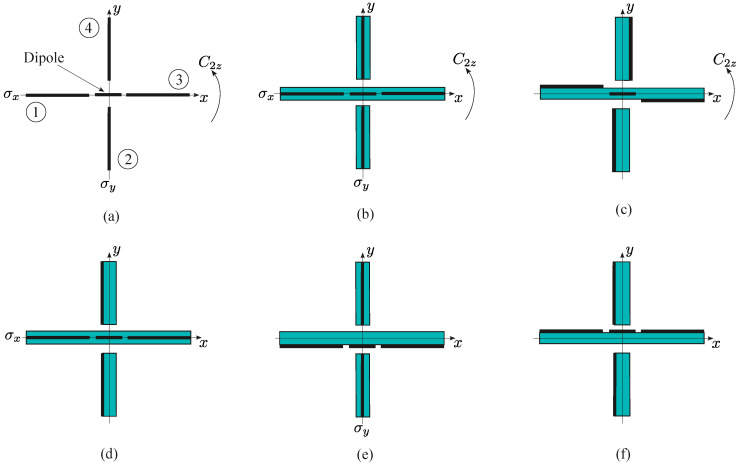
Symmetry of antennas in the x0y plane; the thick lines correspond to graphene layers, and the thin lines delineate the dielectric substrates. (**a**) Free-standing antenna, group $C_{2v}$; the numbers 1 and 3 denote coplanar reflectors with the dipole between them, and numbers 2 and 4 correspond to the orthogonal reflectors; (**b**) antenna with graphene elements between dielectric layers, group $C_{2v}$; (**c**) antenna with reflectors on substrates, group $C_{2}$; (**d**) antenna with the symmetry $C_{s}$ and the plane of symmetry $\sigma_{x}$; (**e**) antenna with the symmetry $C_{s}$ and the plane of symmetry $\sigma_{y}$; (**f**) antenna with the symmetry $C_{1}$, i.e., no symmetry elements.

The symmetry elements of the group $C_{2v}$ are shown in Figure 2a. Symmetry of the discussed antenna can be reduced when we add some dielectric elements to the antenna. The symmetry $C_{2v}$ is preserved if every graphene element is placed between two equal supporting dielectric layers, i.e., it is a sandwich-like element (see Figure 2b).

If the graphene elements of the reflectors are placed on one side of the dielectric substrate, the symmetry is reduced to group $C_{2}$ with only the two-fold rotational axis $C_{2z}$ without planes of symmetry (see Figure 2c). Antennas described by group $C_{s}$ with the plane of symmetry $\sigma_{x}$ and $\sigma_{y}$ are shown in Figure 2d and Figure 2e, respectively. Notice that groups $C_{2z}$ and $C_{s}$ are subgroups of group $C_{2v}$. If all the graphene elements are placed on one side of the substrates, no 2D symmetry is left in the antenna shown in Figure 2f. In this case, however, the horizontal plane of symmetry $\sigma_{z}$ can be present.

### 4.3. Effect of Chemical Potentials on Antenna Symmetry

Symmetry of the antenna system is defined not only by geometry but also by its physical parameters, in particular, by chemical potentials. First, we shall consider the antenna without dielectric substrates in free space with $C_{2v}$ symmetry. With equal chemical potentials on the reflectors, the symmetry is also described by group $C_{2v}$. This symmetry is preserved if the reflectors in each pair of the coplanar and orthogonal reflectors have the same chemical potential. If only one pair of reflectors has the same potential, this yields $C_{s}$ symmetry. If the chemical potentials of all the reflectors are different, the antenna loses all the elements of symmetry $C_{2v}$ (except the unit element *e*, which does not provide any information).

### 4.4. Resulting Symmetry of Antenna

From the point of view of group theory, the resulting symmetry of the structure depends on the combined effect of the dielectric substrates and of the chemical potentials. This can be determined using Curie’s principle of symmetry superposition [33]. This principle states that the symmetry of a complex object is defined by the highest common subgroup of all the groups describing the object. In our case, it is defined by intersection from one side of the group of symmetry of the antenna with substrates with chemical potentials equal to zero, and from the other side, of the group of symmetry of the chemical potentials, for example, $C_{2v}\cap C_{2v}=C_{2v}$, $C_{2v}\cap C_{s}=C_{s}$, $C_{2}\cap C_{s}=C_{1}$.

Notice that with the small thickness of the substrates and low dielectric constants of the dielectric material, the influence of the substrates on the symmetry can be very small, and the resulting symmetry of the antenna will be defined mostly by the chemical potentials. Different resulting symmetries allow us to realize different RPs using the same antenna, as shown by numerical calculations in Section 8.

### 4.5. Symmetry of Currents and Fields: Group $C_{2v}$

The irreducible representations (IRREPs) of group $C_{2v}$ are given in Table 1. The transformations of the graphene currents j, the electric field E, and the magnetic field H are defined by this table as follows. Let us consider, for example, the currents in the coplanar reflector 1 of Figure 1a. Due to the 2D nature of graphene, only two components of the current can exist in the layer, namely, $j_{x}$ and $j_{z}$. The component $j_{z}$ belongs to IRREP $A_{1}$ where all the representations are equal to 1; therefore, after application of all symmetry operators, $j_{z}$ preserves its sign. This means that the $j_{z}$ components in the symmetrical points of the two coplanar (and also in the two orthogonal) reflectors will be the same. On the other side, the component $j_{x}$ belongs to IRREP $B_{1}$; therefore, after rotation by π (the symmetry element $C_{2}$), $j_{x}$ multiplied by the IRREP −1 preserves its modulus but changes the sign. By reflection $\sigma_{x}$, the current $j_{x}$ is not changed (it is the same current in the same graphene reflector). However, after reflection $\sigma_{y}$, one comes from reflector 1 to the symmetrical point in reflector 3 and, multiplying by −1, one has the current in this reflector.

In the case of the electromagnetic fields E and H, one can consider their symmetry at any point of the space. Due to the relation $j=\sigma_{g}E$, where $\sigma_{g}$ is the conductivity of graphene, the symmetry of the electric field E on the graphene elements coincides with the symmetry of the current j.

### 4.6. Symmetry of Currents and Fields: Groups $C_{2}$ and $C_{s}$

The symmetry degeneration Table 2 provides the correspondence between the IRREPs of group $C_{2v}$ and the IRREPs of its subgroups $C_{2}$ and $C_{s}$ [34]. As a result, one can assign different components of current and fields to IRREPs $C_{2}$ or $C_{s}$ using the corresponding IRREPs of group $C_{2v}$. Notice that, in the case of group $C_{s}$, one needs to consider two variants with different orientations of the planes of symmetry separately, namely, one is within the plane of symmetry $\sigma_{x}$ (group $C_{s}^{x}$), and the other within the plane $\sigma_{y}$ (group $C_{s}^{y}$).

For example, the components belonging to the IRREPs $A_{1}$ and $A_{2}$ of $C_{2v}$ will belong to the IRREP *A* of $C_{2}$. However, $A_{1}$ of $C_{2v}$ degenerates to the IRREP *A* of $C_{s}^{x}$, but $A_{2}$ degenerates to the IRREP *B* of $C_{s}^{x}$. The IRREP *A* in the $C_{s}^{x}$ group describes an even symmetry, but the IRREP *B* corresponds to the odd symmetry with respect to the plane $\sigma_{x}$. The presented information allows us to create Table 3, Table 4 and Table 5. Additionally, we present in Table 6 the transformation properties of the currents in the graphene elements as well as the electric and magnetic fields with respect to the plane $\sigma_{z}$.

## 5. Qualitative Analysis of the Currents and Fields

Qualitative analysis of both the near and far fields of the antennas can be completed using the elaborated Table 1 and Table 3, Table 4, Table 5 and Table 6. A special line is the the vertical axis *z* with the parameters x=0 and y=0. Due to the two-fold rotation axis $C_{2}$ in groups $C_{2v}$ and $C_{2}$, the components that are odd with respect to this element (see Table 1 and Table 3, the field components $E_{x}$, $E_{y}$ and also $H_{x}$ and $H_{y}$) are equal to zero on the axis *z* because these components change their sign on the axis. Therefore, the Poynting vector in the *z*-direction is equal to zero, and the antenna does not radiate in this direction. However, groups of symmetry $C_{s}^{x}$ and $C_{s}^{y}$ do not prohibit the existence of the Poynting vector in the *z*-direction. For example, in Table 4, for group $C_{s}^{x}$, the components $E_{x}$ and $H_{y}$ are even with respect to the plane $\sigma_{x}$, i.e., they can exist on the axis *z*, thus providing the Poynting vector in the *z*-direction.

**Figure 3:**
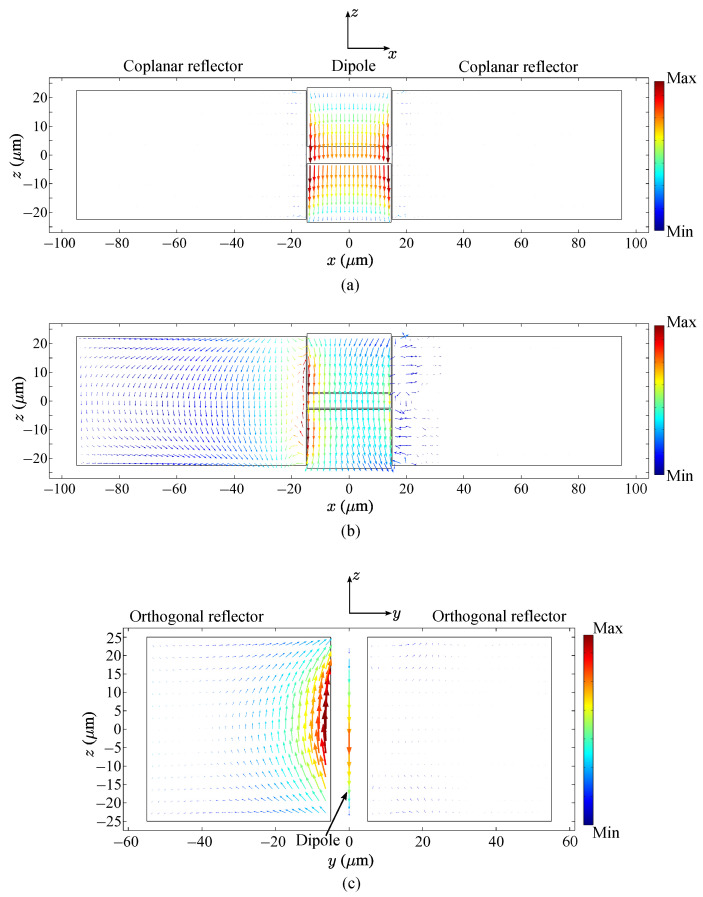
Current distributions on graphene parts at 1.89 THz: (**a**) on the dipole and on the coplanar reflectors (state 0, plane x0z), (**b**) on the dipole and on the coplanar reflectors (state 1, plane x0z), (**c**) on the dipole and orthogonal reflectors (state 2, plane y0z).

**Figure 4:**
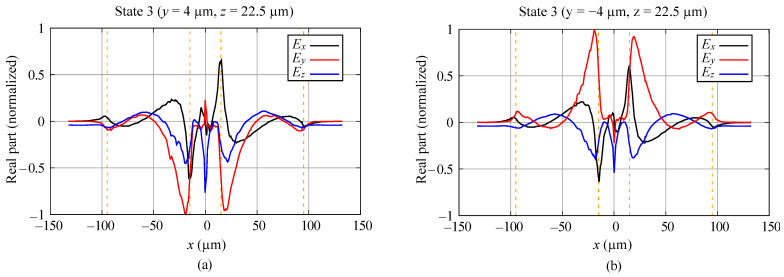
The *x*-dependence of $E_{x}$, $E_{y}$, and $E_{z}$ field components, state 3, f=1.89 THz. The coordinates z=22.5μm, (**a**) y=4μm, (**b**) y=−4μm. The vertical dotted lines delineate the regions of the dipole and the reflectors.

Now, we consider the restrictions produced by the planes of symmetry. If, for example, a current or field component is odd with respect to the plane of symmetry $\sigma_{x}$, it means that this component is zero in this plane. The same is true for the planes $\sigma_{y}$ and $\sigma_{z}$. For example, in Figure 3b, obtained by numerical calculations, one can see that in the plane z=0, the graphene currents $j_{y}=0$ because this current is odd with respect to this plane. The field $E_{x}$ in Figure 4 is odd with respect to the plane $\sigma_{y}$ and, therefore, it is equal to zero in this plane. The argument results obtained by symmetry are exact and can be used as references in numerical calculations.

## 6. Pre-Optimization Design of Graphene Dipole Antenna (Without Reflectors)

Few studies in the literature provide equations for designing terahertz graphene dipole antennas and predicting their plasmonic resonance frequency. In [35], a Fabry–Perot model is presented to estimate the resonance frequency of a graphene-based nano-patch antenna based on its length. That antenna was modeled as an infinitely wide graphene patch suspended in the air, and the results were verified by means of numerical simulations. To take into account the finite width of a realistic graphene-based nano-patch antenna, a partial element equivalent circuit (PEEC) model was developed in [36] to calculate absorption cross-section peaks, which can be used to obtain the device resonance frequencies. That approach is validated using data from [37,38]; however, it does not consider antenna feeding mechanisms. In [39], an RLC resonant circuit model for graphene-based bow-tie antennas was proposed, which was fed by a THz photomixer between its arms. More recently, a circuit model for nanoscale graphene dipole antennas was presented in [40]. Despite permitting prompt physical analysis, the evaluation of antenna performance, and simplified optimization procedures for obtaining a desired set of antenna characteristics, the parameters of antenna circuit models must often be extracted from full-wave simulation data. Furthermore, in addition to the strong influence of the basic graphene parameters (dimensions and chemical potential) and dipole feeding structures, substrate electromagnetic parameters play a fundamental role in defining the operational characteristics of the antenna.

In [41], semi-analytical equations were developed to facilitate the design of rectangular terahertz graphene-based dipole antennas mounted on glass substrates. The equations allow for the direct calculation of the dipole length needed to achieve resonance at a given frequency $f_{r}$, given the antenna width $w_{0}$, graphene chemical potential $\mu_{c0}$, and the dimensions of the feeding structure. The formulation is a combination of the graphene electrostatic scaling law [42,43] and the least squares method [44], which were used with finite-difference time-domain (FDTD) [45,46] simulations, taking into account the dipole feeding mechanism of Figure 1 and various combinations of antenna dimensions and chemical potential values.

A coplanar plate capacitor is formed by the graphene dipole metallic electrodes. Its capacitance (ignoring graphene contribution) is given by $C=\varepsilon_{\mathrm{eff}}w_{0}K(\sqrt{1-k^{2}})/K(k)$, as detailed in [47,48], in which $\varepsilon_{\mathrm{eff}}$ is the effective permittivity around the structure, K(.) is the complete first kind elliptic integral [49], $k=d_{\mathrm{gap}}/L_{s}$, $d_{\mathrm{gap}}$ is the distance between metallic plates, $d_{\mathrm{metal}}$ is the plate width, and $L_{s}=d_{\mathrm{gap}}+2d_{\mathrm{metal}}$. Furthermore, the self-inductance of the metallic electrodes [50] is given by $L=2\cdot 10^{-9}L_{s}\left[\log\left(2L_{s}/w_{0}\right)+0.5+0.2235w_{0}/L_{s}\right].$ Thus, the resonance frequency $f_{m}$, solely regarding the pair of metallic feed contacts composing the graphene dipole, can be approximately calculated by employing the well-known LC circuit equation $f_{m}={\left(2\pi \sqrt{LC}\right)}^{-1}$. Considering the graphene sheets as part of the structure, the resonance frequency $f_{m}$ can be used to calculate the phase contribution of the source and the metallic parts, overall measuring $L_{s}$, which is obtained by $\theta_{s}=\pi f_{r}/f_{m}$, where $f_{r}$ is the graphene dipole first resonance frequency. Thus, the half-cycle contribution $\theta_{g}$ of both graphene sheets in the dipole antenna is $\theta_{g}=\pi -\theta_{s}.$ Therefore, one can see that the total length of the pair of graphene sheets $L_{g}$ is given by $L_{g}=\theta_{g}/\beta ,$ where β is the graphene plasmonic phase constant. Clearly, the total length of the graphene dipole antenna is $l_{0}=L_{s}+L_{g}$.

As is shown in detail by [41], β can be obtained using an adapted version of the quasi-electrostatic scaling parameter [43] for cases in which condition $w_{0}\ll \lambda_{\mathrm{spp}}$ is not met (such as in the cases of the antennas in this paper). The adapted scaling parameter η is given by η=Im[σ(fr)]/(frw0εeff), in which $f_{r}$ is the graphene dipole resonance frequency. In [41], several graphene dipole antennas were simulated using the FDTD method, from which $f_{r}$ has been numerically computed and η has been calculated using the given definition. Finally, it can be shown that $\beta \approx \eta^{a}e^{b}/w_{0},$ in which *e* is Euler’s number and *a* and *b* are functions of $\mu_{c}$ and $w_{0}$ [41]. Thus, since the values of $f_{r}$, $\mu_{c}$, and $w_{0}$ were previously defined, β and $L_{g}$ can be calculated.

For designing a pre-optimization model of our THz graphene dipole antenna, resonating at $f_{r}=1.5$ THz, a high level of efficiency must be assured. It is known that, for low THz applications, the width of graphene nanoribbons should measure tens of micrometers because the imaginary part of the effective refractive index is importantly reduced by increasing the graphene sheet width [51]. As a consequence, the radiation efficiency of the graphene dipole antenna also substantially improves with dipole width in the THz range from approximately 16% to 80%, at resonance frequency, by increasing the dipole width from 2 μm to 32 μm (with $\mu_{c0}=1$ eV and $l_{0}=41\;\mu m$) [41]. For this reason, we have selected the dipole width $w_{0}=30\;\mu m$ for our pre-optimization dipole model. However, $w_{0}$ should be smaller than $l_{0}$ for avoiding undesired width-related resonances. Further fixed parameters in our design are: $d_{\mathrm{gap}}=5\;\mu m$, $d_{\mathrm{metal}}=0.5\;\mu m$, $\epsilon_{r}=3.8$ (glass substrate relative permittivity), and $\mu_{c0}=1.2$ eV. Thus, for our device, by using the given parameters, definitions, and expressions, we obtain $\eta \approx 1.372\times 10^{-10}$, a≈−0.594, b≈−12.722, $\beta \approx 7.129\times 10^{4}$, and $L_{g}=\theta_{g}/\beta \approx 35.4\;\mu m$; finally, the dipole length is estimated to be $l_{0}=\left(d_{\mathrm{gap}}+2d_{\mathrm{metal}}\right)+L_{g}\approx 41.4\;\mu m$ to produce the desired resonance frequency and high radiation efficiency [41]. Finally, note that the term pre-optimization is used in this section due to the fact that the reflectors are not yet being considered at this point. The uncoupled dipole design is optimized, but once reflectors are present and coupled to the active antenna, dipole dimensions along with dimensions of reflectors and chemical potentials of all graphene parts must be optimized for working as a single device. This is explored in the following section.

## 7. Design of Graphene Dipole Antenna with Reflectors

Graphene reflectors are passive elements that can be used to improve and control the gain of a given antenna (such as a graphene dipole). This can be achieved by placing the reflectors at specific distances $d_{i}$ from the dipole, forming a parasitically coupled antenna. Chemical potentials of reflectors can be regulated in order to define maximum gain direction by tuning electromagnetic coupling among the active element (dipole) and passive parts (reflectors). However, the antenna operation spectral range may also be affected due to alterable electromagnetic coupling characteristics. Fortunately, the operation spectral range can be maintained if the chemical potential of the graphene dipole is regulated along with those of the reflectors.

For defining geometric parameters of the antenna in Figure 1b, full-wave numerical electromagnetic simulations were performed using the software CST Studio Suite 2019.In order to minimize computational requirements, graphene elements composing the dipole antenna and reflectors are modeled using a surface impedance boundary condition [9]. The surface impedance boundary condition is commonly used in electromagnetic simulations of graphene sheets to model the behavior of the graphene with interfaces to surrounding media, such as air or dielectric materials. The surface impedance boundary condition allows the representation of graphene as a thin layer of material, i.e., it is represented as a 2D material in a computational mesh. It takes into account the electromagnetic properties of the interface by considering the complex conductivity of the graphene sheet. By defining the surface impedance boundary condition appropriately, the simulation can accurately model the electromagnetic behavior of the thin graphene sheet and the antenna and can predict the performance of the device. The chosen graphene parameters are τ=0.5 ps [52,53] and room temperature T=300 K. As an optimization starting point for defining the device dimensions, the pre-optimization dimensions formerly calculated for the graphene dipole antenna were used ($w_{0}=30\;\mu m$ and $l_{0}=40.4\;\mu m$). Then, reflectors were included in the model. Dimensions of the dipole antenna and reflectors were optimized to preserve the operation band by taking into account the reflectors’ influence (whether they were activated or not). We consider a graphene sheet to be electrically deactivated if its chemical potential is zero and activated otherwise. The dimensions of the graphene elements of the proposed antenna obtained via parametric optimization are shown in Table 7. Note that the operation band of the proposed antenna is chosen to be centered at 1.89 THz, around the second resonance frequency of the dipole, since its radiation efficiency is higher than radiation efficiency seen around $f_{r}=1.5$ THz.

## 8. Numerical Simulations and Equivalent Circuit Analysis

### 8.1. Comparison of Radiation Patterns of the Graphene Dipole Antenna and Quasi-Omnidirectional Antenna with Reflectors

**Figure 5:**
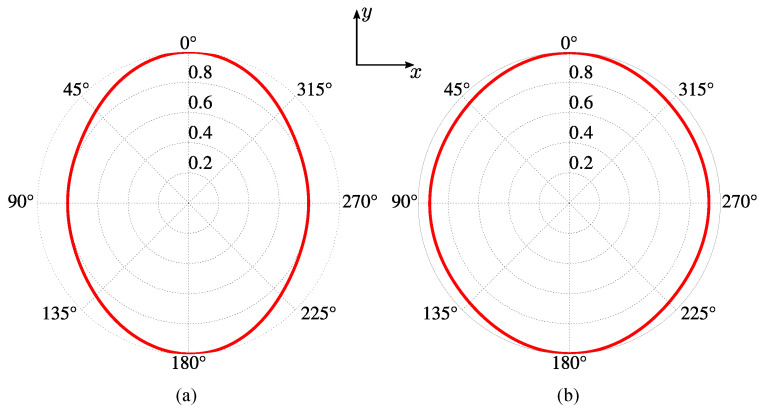
The normalized RP (red curves) in the x0y plane: (**a**) dipole antenna with the chemical potential of the dipole $\mu_{c0}=0.55$ eV and the chemical potentials of all the reflectors equal to 0, i.e., $\mu_{c}^{r_{1}}=\mu_{c}^{r_{2}}=\mu_{c}^{r_{3}}=\mu_{c}^{r_{4}}=0$ eV, (**b**) dipole with the chemical potential $\mu_{c0}=0.55$ eV and the chemical potentials of the reflectors $\mu_{c}^{r_{2}}=\mu_{c}^{r_{4}}=0$ eV and $\mu_{c}^{r_{1}}=\mu_{c}^{r_{3}}=0.11$ eV. The RPs in both cases were calculated at the frequency 1.89 THz.

Symmetry of a common linear wire antenna oriented in the *z*-direction, in the plane x0y, is described by the continuous group $C_{\infty v}$ with the axis $C_{z}$ of the infinite order. This symmetry provides omnidirectional RP. The graphene antenna without reflectors is, in fact, a rectangular one with a one-atom thickness. Symmetry of this antenna in the x0y plane is $C_{2v}$, which is a subgroup of $C_{\infty v}$. As a result of symmetry reduction, the corresponding RP is not omnidirectional. The elliptic-like diagram of such a graphene dipole antenna is shown in Figure 5a. The geometric dimensions of the antenna in this example are given in Section 7.

As a first demonstration of the effect of the reflectors in RP, we show that it is possible to transform the elliptic-like RP of the graphene antenna in Figure 5a to the quasi-omnidirectional one by applying certain chemical potentials on the reflectors. With $\mu_{c}^{r_{2}}=\mu_{c}^{r_{4}}=0$ eV and $\mu_{c}^{r_{1}}=\mu_{c}^{r_{3}}=0.11$ eV on the reflectors, the RP is transformed to an almost circular RP, as shown in Figure 5b. Quantitatively, the ratio of the minor axis to the major one for the antenna without reflectors is 0.8, but this parameter for the antenna with reflectors increases to 0.93.

### 8.2. Operation States and Characteristics of the Antenna

The activation setup of the reflectors defines the following six antenna operation states:

(state 0) coplanar and orthogonal reflectors are electrically deactivated ($\mu_{c}^{r_{i}}=0$ eV, for 1≤i≤4), which gives the symmetry elements of the antenna $C_{2z}$ as $\sigma_{x}$ and $\sigma_{y}$;

(state 1) a single coplanar reflector is exclusively activated ($\mu_{c}^{r_{1}}=1.2$ eV and $\mu_{c}^{r_{3}}=0$ eV, or vice versa), and in this case the symmetry element is $\sigma_{x}$;

(state 2) only one orthogonal reflector is activated ($\mu_{c}^{r_{4}}=0.4$ eV and $\mu_{c}^{r_{2}}=0$ eV, or vice versa), and the symmetry element is $\sigma_{y}$;

(state 3) the pair of coplanar reflectors is uniquely activated ($\mu_{c}^{r_{1}}=\mu_{c}^{r_{3}}=0.6$ eV), and the symmetry elements are $C_{2z}$, $\sigma_{x}$, and $\sigma_{y}$;

(state 4) activation is limited to the pair of orthogonal reflectors ($\mu_{c}^{r_{2}}=\mu_{c}^{r_{4}}=0.4$ eV), and the symmetry elements are $C_{2z}$, $\sigma_{x}$, and $\sigma_{y}$;

(state 5) coplanar reflectors ($\mu_{c}^{r_{1}}=\mu_{c}^{r_{3}}=1.2$ eV) and one orthogonal reflector ($\mu_{c}^{r_{2}}=0.5$ eV or $\mu_{c}^{r_{4}}=0.5$ eV) are activated, and the symmetry element is $\sigma_{y}$.

The antenna can also operate in the beam steering regime (BSR), where only one coplanar reflector and one orthogonal reflector are properly activated, with no elements in 2D symmetry. For instance, the main lobe azimuth angle is set to $45^{\circ }$ when $\mu_{c}^{r_{1}}=1.2$ eV and $\mu_{c}^{r_{4}}=0.3$ eV.

In state 1 and BSR, the dipole chemical potential $\mu_{c0}$ is set to 0.4 eV and, in state 4, $\mu_{c0}=0.8$ eV. For all other states, $\mu_{c0}=1.2$ eV. This correction of $\mu_{c0}$ is required for preserving the central frequency of the antenna $f_{c}=1.89$ THz.

**Figure 6:**
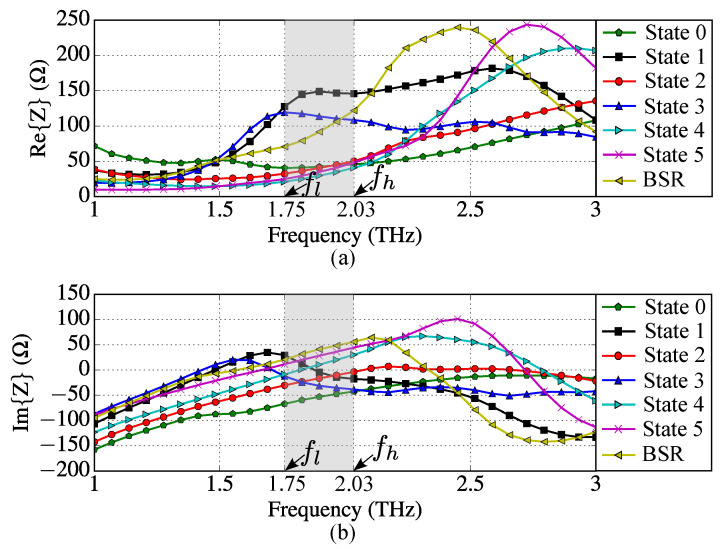
Antenna input impedances for the six states and BSR: (**a**) resistances and (**b**) reactances. The gray shadow defines the operation band ($f_{c}=1.89$ THz).

**Figure 7:**
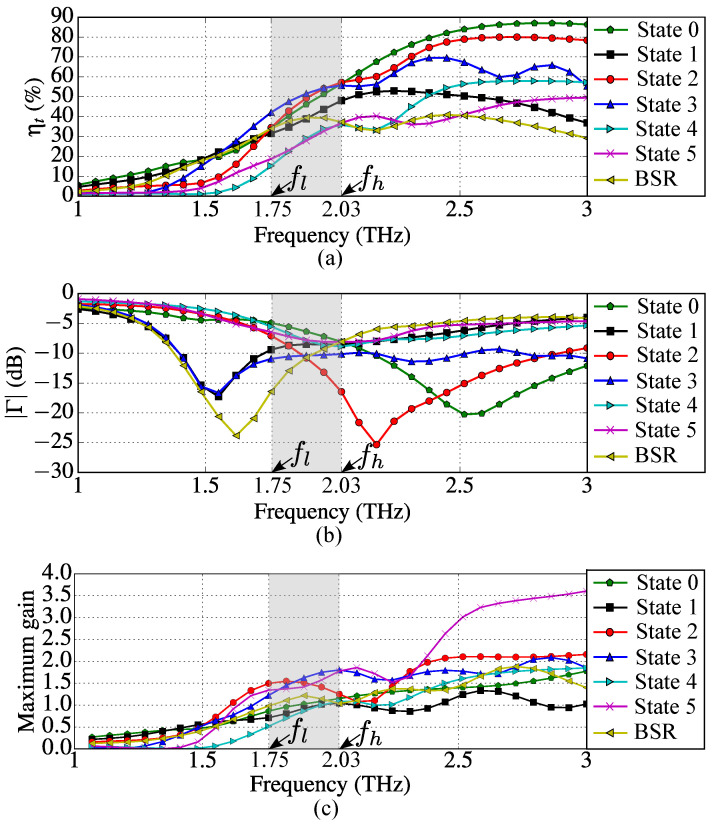
Antenna characteristics for each of the six operation states and BSR as functions of frequency: (**a**) total efficiencies $\eta_{t}$ and (**b**) magnitudes of reflection coefficients Γ (dB) and (**c**) maximum gains. The gray shadow defines the operation band ($f_{c}=1.89$ THz).

Figure 6 shows antenna input impedances *Z*, while Figure 7 demonstrates total efficiency $\eta_{t}$, magnitude of input reflection coefficients Γ and maximum gains calculated for each device operation state and BSR from 1 THz to 3 THz. The antenna operating range of frequencies was defined such that |Γ|≤−5 dB (see Figure 7b). In Figure 6 and Figure 7, the operation band $\left[f_{l},f_{h}\right]$ is highlighted (it is valid for all operation states and BSR). Since the lower frequency of the band is $f_{l}=1.75$ THz and the higher frequency is $f_{h}=2.03$ THz, the antenna fractional bandwidth is $BW\left(\%\right)=200\left(f_{h}-f_{l}\right)/\left(f_{l}+f_{h}\right)\approx 14.8\%$.

The parameters Γ and $\eta_{t}$ were calculated using as the reference impedance $Z_{0}$ the arithmetic mean of the input resistances obtained at f=1.8 THz for all states and BSR. The calculated impedance $Z_{0}=67.6$ Ω can be obtained using the photomixer described in [30] by properly setting the chemical potential of its graphene emitter.

**Figure 8:**
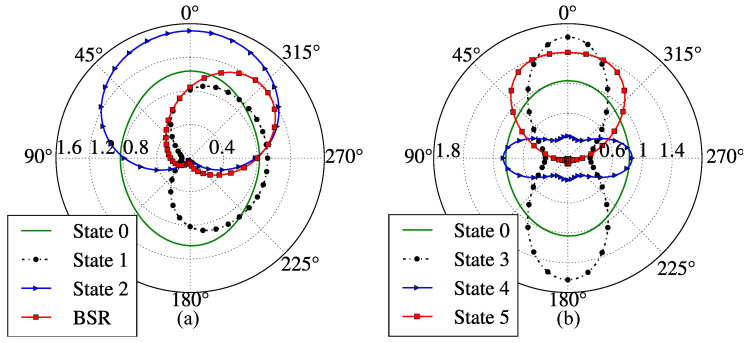
Antenna radiation patterns at $f_{c}=1.89$ THz on azimuth plane x0y: (**a**) for states 0 (reference), 1, 2, and BSR, (**b**) states 0 (reference), 3, 4, and 5.

The RPs of the proposed antenna on azimuth plane x0y for the fixed states and BSR are depicted in Figure 8.

In state 0, by deactivating coplanar and orthogonal reflectors, the antenna operates in its non-directional state, as expected due to partial transparency of deactivated graphene. The diagram is slightly different from the omnidirectional one due to the absence of full rotational symmetry around the *z*-axis (see Section 8.1).

The antenna diagram for state 1 has its main lobe with maximum radiation directed parallel to versor $-\hat{x}$ (see Figure 8a and Table 8). Thus, state 1 is a directive one. By permuting the chemical potentials of the coplanar reflectors, the main radiation lobe can be flipped by $180^{\circ }$, i.e., maximum radiation is aligned with $\hat{x}$. For state 2, the main lobe is oriented as $\hat{y}$ due to the conductivity of the activated graphene orthogonal reflector ($\mu_{c}^{r_{4}}=0.4$ eV), as seen in Figure 8a and Table 8. Similarly to state 1, the main lobe can be directed along $-\hat{y}$ by permuting the chemical potentials of the orthogonal reflectors.

For states 3 and 4, the radiation lobes are narrower (i.e., with lower half-power beam width HPBW) along the *x*- and *y*-axes, as illustrated by Figure 8b. This is due to the simultaneous activation of coplanar reflectors in state 3 and orthogonal reflectors in state 4.

Similarly, for state 5, due to the simultaneous activation of coplanar reflectors and an orthogonal reflector, the main radiation lobe is oriented parallel to $\hat{y}$ and is more directive than in state 2 (see Figure 8).

In BSR, due to the simultaneous activation of an orthogonal reflector and a coplanar reflector, the main lobe can be rotated by $45^{\circ }$ with respect to $\pm \hat{y}$. Thus, the antenna in this regime has its main radiation lobe with the azimuth angle of 45${}^{\circ }$ measured from the *y*-axis when $\mu_{c}^{r_{4}}=0.3$ eV and $\mu_{c}^{r_{1}}=1.2$, as demonstrated in Figure 8a. Conversely, by activating an orthogonal reflector ($\mu_{c}^{r_{2}}=0.3$ eV) and a coplanar reflector ($\mu_{c}^{r_{1}}=1.2$ eV), the main lobe is rotated by $45^{\circ }$ with respect to versor $\hat{y}$.

Table 8 summarizes the fundamental parameters, the symmetry, and the respective 3D RPs of the proposed device calculated for all states at the central frequency $f_{c}=1.89$ THz. The highest values of $\eta_{t}$ and gain are produced in state 3, while the highest value of the front-to-back ratio (FBR) is produced in state 5. FBR is the ratio of maximum gain in the main radiation lobe and the maximum gain in the opposite direction. The minimum of |Γ| is observed in state 2.

**Figure 9:**
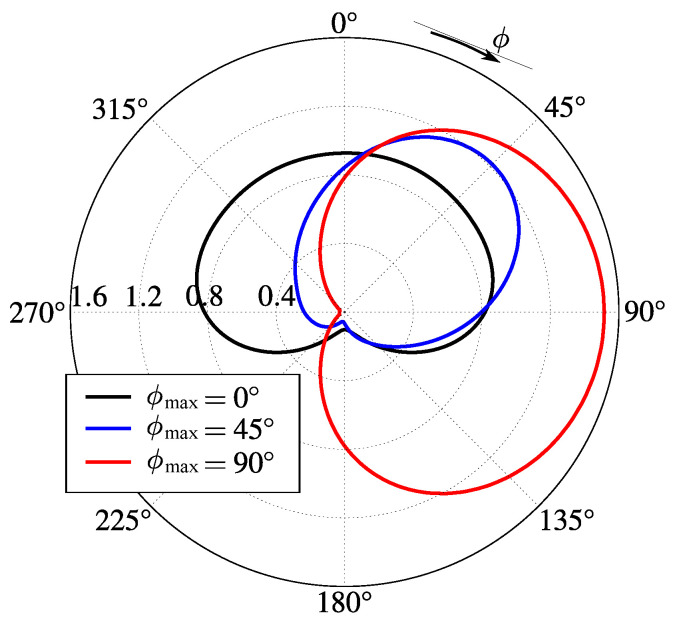
Radiation patterns of the proposed device in free space illustrating lobe rotation capabilities: $\phi_{\max}$ is the angle of maximum gain.

Finally, for the sake of illustration of lobe rotation capabilities of the proposed device, we present in Figure 9 three radiation patterns with maximum gain angle $\phi_{\max}$ set to $0^{\circ }$, $45^{\circ }$, and $90^{\circ }$. The necessary chemical potentials for each graphene part of the antenna for producing the lobes shown are given in Table 9. Note that the $360^{\circ }$ main lobe rotation is possible due to the demonstrated symmetry properties of the device.

### 8.3. Near Field in the Antenna

**Figure 10:**
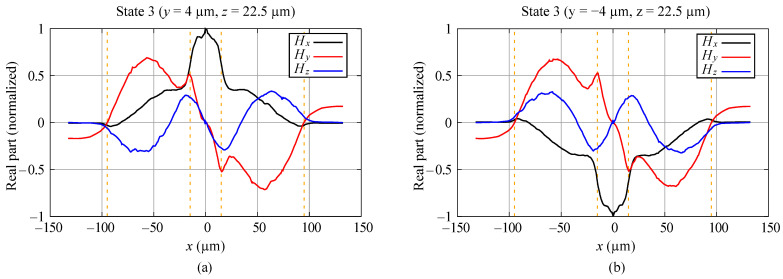
The *x*-dependence of $H_{x}$, $H_{y}$, and $H_{z}$ field components, state 3, f=1.89 THz. The coordinates z=22.5μm, (**a**) y=4μm, (**b**) y=−4μm. The vertical dotted lines delineate the regions of the dipole and the reflectors.

The proposed graphene antenna electromagnetic field distribution is distinct from that of the metal wire dipole because of its different composition and geometry. Here, we consider the antenna with symmetry $C_{2v}$. In order to discuss the peculiarities of the field, we calculated the distribution of the fields along the line oriented in the *x*-direction for state 3, f=1.89 THz. The line is fixed at the coordinate z=22.5μm. The components $E_{x}$, $E_{y}$, and $E_{z}$ at y=4μm are presented in Figure 4a and at y=−4μm in Figure 4b. The components $H_{x}$, $H_{y}$, and $H_{z}$ for the same coordinates are given in Figure 10a,b.

Analyzing these graphics, one can see that the symmetry of the field components is in accordance with the results of the group-theoretical predictions in Section 4.5 and the discussion in Section 5. Small discrepancies in the absolute values of the fields in the symmetric points are due the limits in accuracy of the numerical method.

The near field in the antenna contains all components of the electric and magnetic fields of high intensity, which vary rapidly in space. The presence of the reflectors where the excited currents produce the fields significantly changes the structure of the near field in comparison with the antenna without reflectors (not presented here). The components $E_{z}$ and $H_{y}$ (more specifically, $E_{\theta }$ and $H_{\phi }$) for |x|>95μm provide the Poynting vector of the outgoing wave. Notice that the components of the resonant near fields in the region of the antenna and the reflectors are much higher than the corresponding components of the radiated field.

### 8.4. Circuit Representation of Graphene Dipole Antenna with Reflectors

Circuit representations of the antenna with its reflectors can be used to study the frequency characteristics of the antenna. As demonstrated in [5], a graphene dipole antenna can be represented by an RLC circuit fed by an electric source representing the dipole photomixer excitation. In [54], asynchronously tuned coupled-resonator circuits are presented for analysis of electromagnetically coupled resonators that may resonate at different frequencies. This is the same case as the proposed graphene antenna when one of the reflectors is activated.

A graphene patch surface impedance is defined by $Z_{p}\left(\omega \right)=1/\left(N\sigma_{g}\left(\omega \right)\right)$, where $\sigma_{g}\left(\omega \right)$ is given by (1) and *N* is the number of graphene layers composing the patch [9]. Notice that $\sigma_{g}\left(\omega \right)$ is dependent on the graphene electrochemical potential, which is specifically set for each graphene patch depending on the proposed device operating state (see Table 8). Thus, the graphene resonance frequency depends on sheet dimensions, on $\sigma_{g}\left(\omega \right)$, and on the surrounding media parameters [9,41].

**Figure 11:**
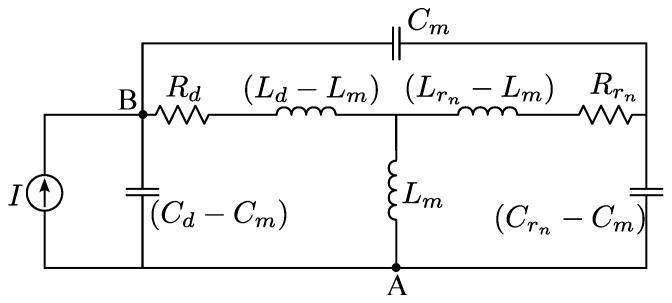
Circuit representation of the graphene dipole antenna coupled with the activated graphene reflector placed near the dipole.

The graphene dipole and the activated graphene reflector act as electromagnetically coupled resonators, resonating at different frequencies. Therefore, a suitable equivalent circuit representation [54] for the proposed graphene antenna in Figure 1b, with only one activated graphene reflector, is depicted by Figure 11. The graphene dipole circuit elements are $R_{d}$ (resistance of graphene dipole), $L_{d}$ (dipole inductance), and $C_{d}$ (dipole capacitance). Furthermore, $R_{r_{n}}$, $L_{r_{n}}$, and $C_{r_{n}}$ are, respectively, the equivalent resistance, inductance, and capacitance associated with the discussed graphene reflector. Circuit parameters $C_{m}$ and $L_{m}$ are coupling capacitance and coupling inductance established respectively by electric and magnetic field coupling between the dipole and the activated graphene reflector. Finally, the graphene dipole terminals are associated with nodes A and B in the circuit of Figure 11. A photomixer is used to convert optical signals to electrical current, which feeds the antenna [30]. Thus, the current source represents the optically-induced photomixer current.

In order to estimate circuit lumped parameters, we suppose that our device is operating in state 1. For this specific case, only a coplanar reflector is activated (i.e., $\mu_{c}^{r_{1}}=1.2$ eV), and the graphene dipole chemical potential is set to $\mu_{c0}=0.4$ eV.

Since the circuit model is based on the idea of two electromagnetically coupled resonators [54], we initially simulate the dipole antenna with our full-wave model considering all reflectors deactivated (their chemical potentials are set to zero), i.e., the dipole is practically decoupled from the reflectors, and we set $\mu_{c0}=0.4$ eV. This condition allows us to estimate the lumped circuit parameters for the dipole ($R_{d}$, $L_{d}$ and $C_{d}$) when it is decoupled from all reflectors. Analyzing the circuit in Figure 11, one can see that the dipole circuit is decoupled from the reflector when $R_{r_{1}}\to \infty$, $L_{r_{1}}\to 0$, $C_{r_{1}}\to 0$, $C_{m}\to 0$, and $L_{m}\to 0$. The aforementioned decoupling conditions are obtained when the graphene conductivity of the reflector is small, since $\mu_{c}^{r_{1}}=0$.

**Figure 12:**
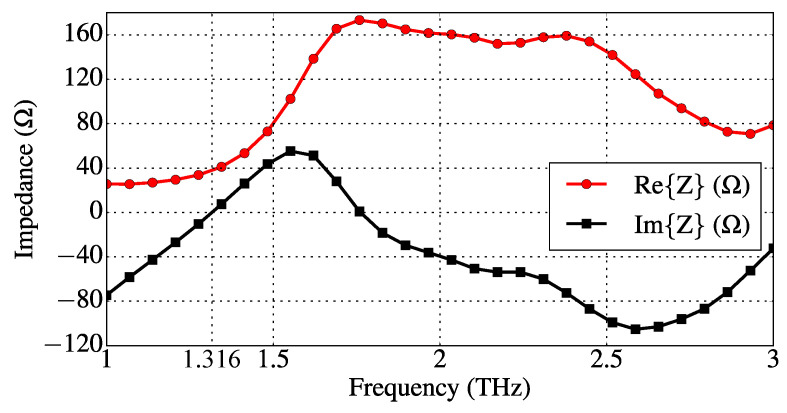
Graphene dipole impedance obtained via full-wave CST simulation (dipole with $\mu_{c0}=0.4$ eV and deactivated reflectors). The dipole first resonance frequency is $f_{1}^{d}=1.316$ THz, at which Re{Z}$(f_{1}^{d})=37.9\;\Omega$ and Im{Z}$(f_{1}^{d})=0$.

The full-wave dipole simulation provides the impedance shown in Figure 12, from which we see that the decoupled dipole’s first resonance frequency is $f_{1}^{d}\approx 1.316$ THz. At the frequency $f_{1}^{d}$, we obtain, from Figure 12, $R_{d}\;\approx$ Re{Z}$(f_{1}^{d})\approx 37.9\;\Omega$. Furthermore, in order to obtain the dipole circuit parameters $L_{d}$ and $C_{d}$, we extract the values of Im{Z} obtained at the frequencies around $f_{1}^{d}$. At $f_{L}=1.25$ THz and $f_{H}=1.35$ THz, one has Im{Z}$(f_{L})\approx -16.27\;\Omega$ and Im{Z}$(f_{H})\approx 8.89\;\Omega$. Therefore, we may write two circuital equations: $\omega_{L}L_{d}-\omega_{L}^{-1}C_{d}^{-1}=-16.27$ and $\omega_{H}L_{d}-\omega_{H}^{-1}C_{d}^{-1}=8.89$, where $\omega_{L}=2\pi f_{L}$ and $\omega_{H}=2\pi f_{H}$. By solving the above-formed linear system for $L_{d}$ and $C_{d}^{-1}$, one gets $L_{d}\approx 1.98\times 10^{-11}$ H and $C_{d}\approx 7.41\times 10^{-16}$ F.

**Figure 13:**
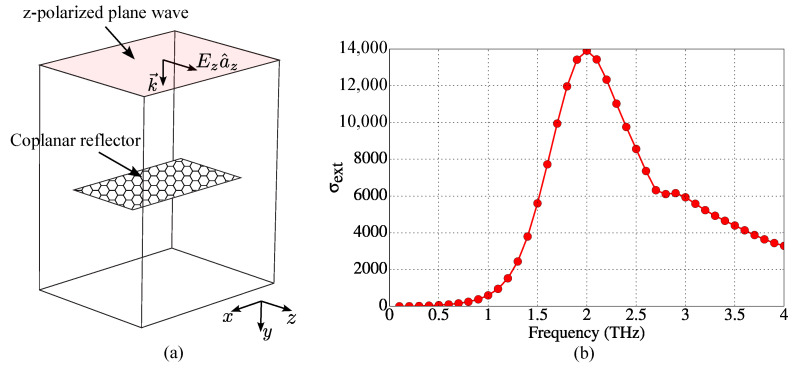
Numerical calculation of resonance frequency of graphene coplanar reflector in free space: (**a**) simulation geometry and (**b**) extinction cross-section of the reflector ($\mu_{c}^{r_{1}}=1.2$ eV). Coplanar reflector resonance frequency is $f_{1}^{r_{1}}=2.0$ THz.

Decoupled activated coplanar reflector lumped parameters can be estimated by determining the reflector resonance frequency $f_{1}^{r_{1}}$. In this work, we employ the procedure described in [37] for numerically calculating a finite-length graphene plate extinction cross-section $\sigma_{\mathrm{ext}}$, whose peak is associated with the sheet resonance frequency. The numerical procedure starts by simulating the graphene sheet excited by a plane wave, as illustrated by Figure 13a. Then, ratios of surface integrals involving Poynting vectors of the total, scattered, and incident fields are numerically computed [37], producing, in our case, the curve shown in Figure 13b, in which we see that $f_{1}^{r_{1}}\approx 2.0$ THz. It is important to point out that the *z*-polarization of plane wave is so defined for agreeing with the stronger current direction of the coupled coplanar reflector, as shown in Figure 3b. In order to estimate the lumped circuit parameters for the coplanar reflector, we use the calculated values of the dipole lumped parameters as a reference. Because the coplanar reflector length is greater than the dipole dimensions, we estimate that $L_{r_{1}}\approx 2.45L_{d}\approx 4.851\times 10^{-11}$ H. This estimation provides $C_{r_{1}}=\left[1/{\left(2\pi f_{1}^{r_{1}}\right)}^{2}\right]/L_{r_{1}}\approx 1.305\times 10^{-16}$ F. Furthermore, since the chemical potential is higher on the reflector in state 1, we estimate that $R_{r_{1}}\approx R_{d}/5$.

**Figure 14:**
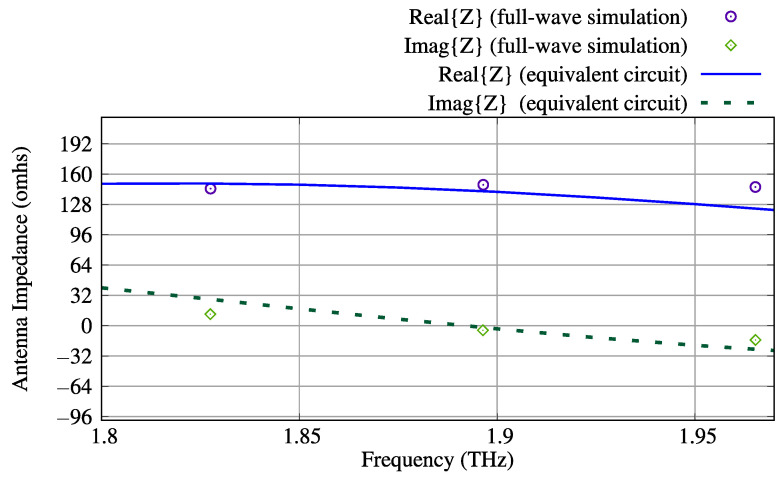
Antenna impedance obtained via full-wave CST simulation of graphene dipole with an activated coplanar graphene reflector (state 1) and the impedance of its equivalent circuit.

Finally, the electromagnetic coupling circuital parameters $L_{m}$ and $C_{m}$ were obtained in this work by manual parametric optimization. We found that $L_{m}\approx 1.20L_{d}\approx 2.376\times 10^{-11}$ H and $C_{m}\approx 0.80C_{d}\approx 5.928\times 10^{-16}$ F. By comparing the full-wave antenna impedance for the electromagnetically coupled dipole-reflector device with the equivalent circuit impedance, we observed that $R_{d}$ should be slightly reduced from 37.9Ω to $R_{d}=33.0\;\Omega$ for better fitting. Figure 14 shows a comparison between the antenna impedance while operating in state 1 (the graphene dipole and the activated coplanar reflector are electromagnetically coupled), obtained by means of the full-wave CST simulation, and the impedance of its equivalent circuit obtained with the calculated lumped circuit parameters. As one can see in Figure 14, the procedure suggested in this work for estimating the lumped circuit parameters produced a good agreement between the full-wave and equivalent circuit models.

### 8.5. Effect of Substrates and Base and θ-Control of RP

The above analysis of the antenna was completed for the case of absence of any substrate. As a result, due to the plane of symmetry $\sigma_{z}$, for example, the maximum of the RP is always situated in the x0y plane. In practice, the graphene reflectors are placed on substrates, and the antenna as a whole is placed on a supporting base. These dielectric elements modify the parameters and RP of the antenna.

**Figure 15:**
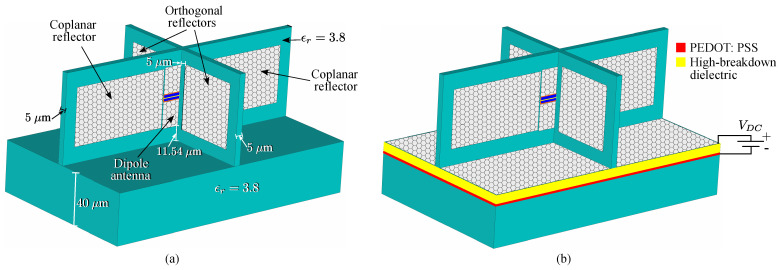
Antenna (**a**) with dielectric substrates and dielectric base, where a system of the chemical potential control is not shown; (**b**) with additional graphene layer on the base and the base control system. Navy blue represents the dielectric material.

One possible practical realization of the proposed antenna is shown in Figure 15. The coplanar reflectors, dipole, and the orthogonal reflectors are placed on ${\mathrm{SiO}}_{2}$ substrates with relative permittivity $\epsilon_{r}=3.8$ and thickness 5μm. The whole structure is mounted on a ${\mathrm{SiO}}_{2}$ base infinite in *x*- and *y*-directions with thickness 40μm. Because of the presence of the dielectric substrates and the base, as well as the applied chemical potentials, all possible symmetry elements of the antenna in this case are absent.

**Figure 16:**
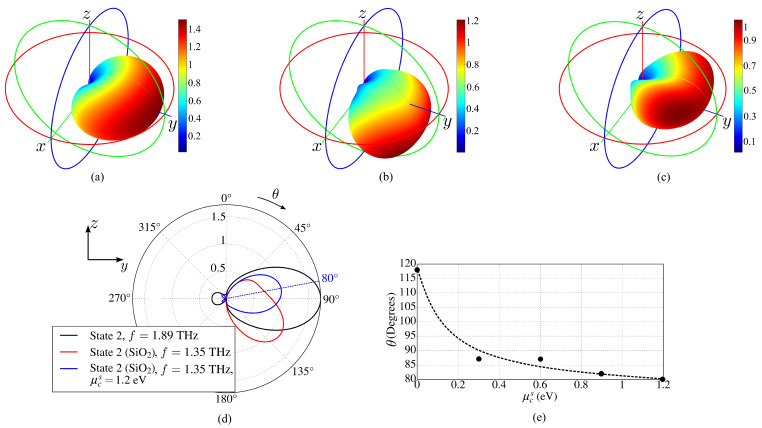
Comparison of antenna radiation patterns in state 2: (**a**) in free space, (**b**) with dielectric supporting substrates for reflectors and with dielectric base, (**c**) the dielectric base is covered by an additional graphene layer with $\mu_{c}^{s}=1.2$ eV, gain is mapped by the color bars, (**d**) RPs in the plane y0z for the cases (**a**–**c**), (**e**) $\mu_{c}^{s}$ dependence of the RP inclination angle θ (the points are the result of numerical calculations).

Figure 16a shows the radiation patterns of the antenna operating in free space, and Figure 16b shows it with ${\mathrm{SiO}}_{2}$ base and substrates.

For the free space case, the radiation pattern is calculated at $f_{c}=1.89$ THz (see Table 1, state 2) and, when dielectric elements are included, $f_{c}=1.35$ THz. The thin reflector substrates have a relatively small influence on the antenna characteristics. However, the resonance frequency lowers due to the Purcell effect and, as expected [55] due to the optically denser massive dielectric base, the main radiation lobe tilts towards the base with $\theta =117^{\circ }$, as can be seen in Figure 16b,d. Figure 16d,e demonstrate that it is possible to correct the angle θ by using the chemical potential $\mu_{c}^{s}$ of the additional graphene layer placed on the base.

## 9. Discussion

The wavelength in free space for the frequency f=1.89 THz is λ≈160μm. The length of the graphene dipole $l_{0}=46.92\;\mu m$ is 3.4 times less than λ. The volume $l_{x}\times l_{y}\times l_{z}$ occupied by the discussed antenna in physical space is 160.66μm ×110μm ×50μm. Thus, the highest dimension of the antenna is defined by the reflectors. It is approximately equal to the free-space wavelength λ.

Now, we shall provide several comments on the presented material. First, the control of the chemical potential in every graphene element can be fulfilled by applying a tunable DC voltage $V_{DC}$ between the graphene sheet and a thin polysilicon layer [56] embedded in the dielectric substrate. This layer with relatively high conductivity is used as a gate electrode [57,58]. The thickness of the layer is very small, and it was shown that its effect on the parameters of the corresponding device is negligible [59]. Therefore, we did not include this element in our model. A choice of the dielectric material between graphene and the gate electrode (such as, for example, $HfO_{2}$, $TiO_{2}$, $Al_{2}O_{3}$, and ion gel gate dielectrics) allows one to change the Fermi energy up to 1.3 eV without voltage breakdown [60]. An example of the graphene layer biased by the electric field is given in Figure 15b. Notice that the same scheme can also be used for biasing of the dipole and reflector graphene sheets of Figure 1 (for simplicity, it is not shown in Figure 15b). Some other problems of graphene technology are discussed in recent review papers [61,62].

Secondly, applying a variable voltage to the coplanar and orthogonal reflectors in the beam steering regime (BSR) in the form of, for example, $\sin(\Omega_{s}t)$ and $\cos(\Omega_{s}t)$, where $\Omega_{s}$ is a sweeping frequency and *t* is time, one can provide a continuous angular rotation of the antenna beam, maintaining reasonable levels of the principal parameters of the antenna.

Thirdly, the suggested antenna can be optimized. The optimization parameters can be the geometrical dimensions of the active dipole antenna, the dimensions of the reflectors, the gaps between the dipole and reflectors, the number of graphene layers, the chemical potentials of the dipole and the reflectors, and the dimensions of the dielectric substrates and their physical parameters. Depending on application of the antenna, the objective function (or functions) can be different for example, the parameters FBR, gain, |Γ|, $\eta_{t}$, the antenna bandwidth, and the beam width. Thus, it can be a multi-objective and a multi-parametric optimization problem which requires special consideration. This is planned in future work.

The group-theoretical approach developed in our work allows one to reduce the volume of numerical calculus. For example, in the case of $C_{2v}$ symmetry, it is sufficient to consider only one-eighth of the whole physical space. Such a reduction allows one to greatly accelerate the optimization process of the antenna.

## 10. Conclusions

In this paper, a novel smart graphene antenna with a simple design and dynamic control of its characteristics was suggested and analyzed. Group-theoretical analysis of the antenna presented in this work allows one to predict the main properties of the radiation diagram of the antenna without solving the corresponding boundary-value problem and also to reduce the volume of numerical calculations.

We have shown that, by tuning chemical potentials of the graphene reflectors and the graphene dipole, one can change the antenna radiation pattern, preserving the antenna operation band. In our work, we have calculated the main parameters of the antenna such as input reflection coefficient, total efficiency, front-to-back ratio, and gain for different operation states of the antenna. Due to the vertical orientation of the dipole with respect to the dielectric base and the presence of the four reflectors, the antenna can provide $360^{\circ }$ beam steering of its radiation pattern. Also, with an additional graphene layer on the base, a certain control of the radiation pattern in the θ-direction is possible. Additionally, an equivalent circuit of the antenna for analysis of its frequency characteristics is suggested. Many numerical calculations confirm the presented theoretical results.

One of the key contributions of our proposed graphene antenna is its ability to achieve continuous 360${}^{\circ }$ beam steering on the azimuth plane and also continuous beam steering on elevation plane. This feature sets our antenna apart from existing THz antennas, as it allows for seamless and uninterrupted beam control over the entire azimuthal range. The ability to steer the beam continuously provides enhanced flexibility and adaptability in a wide range of applications. For instance, in THz communication systems, the antenna can dynamically track a moving receiver or transmitter, maintaining a stable and robust link. Furthermore, since THz waves can penetrate certain materials, providing valuable information in fields like medical imaging and security screening, continuous beam steering allows for more efficient scanning and mapping of the target area, enabling quick and automatic data acquisition along with improved imaging resolution.

In future work, we intend to perform optimization of radiation efficiency as well as other parameters and characteristics of the proposed device. We hope that the suggested antenna can find versatile applications.

## Figures and Tables

**Table 1:** IRREPs of group $C_{2v}$; current and field transformations.

C2v	*e*	$C_{2}$	$\sigma_{x}$	$\sigma_{y}$	Current j	Field E	Field H
$A_{1}$	1	1	1	1	$j_{z}$	$E_{z}$	
$A_{2}$	1	1	−1	−1			$H_{z}$
$B_{1}$	1	−1	1	−1	$j_{x}$	$E_{x}$	$H_{y}$
$B_{2}$	1	−1	−1	1	$j_{y}$	$E_{y}$	$H_{x}$

**Table 2:** Symmetry degeneration table of group $C_{2v}$.

C2v	$C_{2}$	$C_{s}^{x}$	$C_{s}^{y}$
$A_{1}$	*A*	*A*	*A*
$A_{2}$	*A*	*B*	*B*
$B_{1}$	*B*	*A*	*B*
$B_{2}$	*B*	*B*	*A*

**Table 3:** IRREPs of group $C_{2}$; current and field transformations.

C2	*e*	$C_{2}$	Current j	Field E	Field H
*A*	1	1	$j_{z}$	$E_{z}$	$H_{z}$
*B*	1	−1	$j_{x}$, $j_{y}$	$E_{x}$, $E_{y}$	$H_{x}$, $H_{y}$

**Table 4:** IRREPs of group $C_{s}^{x}$, plane of symmetry $\sigma_{x}$; current and field transformations.

Csx	*e*	$\sigma_{x}$	Current j	Field E	Field H
*A*	1	1	$j_{x}$, $j_{z}$	$E_{x}$, $E_{z}$	$H_{y}$
*B*	1	−1	$j_{y}$	$E_{y}$	$H_{x}$, $H_{z}$

**Table 5:** IRREPs of group $C_{s}^{y}$, plane of symmetry $\sigma_{y}$; current and field transformations.

Csy	*e*	$\sigma_{y}$	Current j	Field E	Field H
*A*	1	1	$j_{y}$, $j_{z}$	$E_{y}$, $E_{z}$	$H_{x}$
*B*	1	−1	$j_{x}$	$E_{x}$	$H_{y}$, $H_{z}$

**Table 6:** IRREPs of group $C_{s}^{z}$, plane of symmetry $\sigma_{z}$; current and field transformations.

Csz	*e*	$\sigma_{z}$	Current j	Field E	Field H
*A*	1	1	$j_{z}$	$E_{z}$	$H_{x}$, $H_{y}$
*B*	1	−1	$j_{x}$, $j_{y}$	$E_{x}$, $E_{y}$	$H_{z}$

**Table 7:** Optimized dimensions of graphene elements of the proposed antenna.

Parameter	Dimension (μm)	Parameter	Dimension (μm)
$w_{0}$	29.32	$w_{r_{2}}$	50
$l_{0}$	46.92	$w_{r_{4}}$	50
$l_{r_{1}}$	45	$l_{r_{2}}$	50
$l_{r_{3}}$	45	$l_{r_{4}}$	50
$w_{r_{1}}$	80	$d_{x}$	0.33
$w_{r_{3}}$	80	$d_{y}$	5

**Table 8:** Fundamental parameters and characteristics of the antenna at the central frequency 1.89 THz.

State	FBR	Maximum Gain	|Γ| (dB)	$\eta_{t}$ (%)	Chemical Potential of Reflectors	HPBW ($\theta =90^{\circ }$)	Symmetry Elements	Radiation Pattern	
0	1	1.04	−6.38	46.2	$\mu_{c}^{r_{1}}=\mu_{c}^{r_{2}}=$ $\mu_{c}^{r_{3}}=\mu_{c}^{r_{4}}=0$ eV, $\mu_{c0}=1.2$ eV	–	$C_{2z}$, $\sigma_{x}$, $\sigma_{y}$	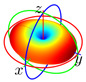	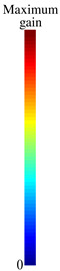
1	9.73	0.93	−8.49	38.9	$\mu_{c}^{r_{1}}=1.2$ eV, $\mu_{c}^{r_{3}}=0$ eV, (flipped lobe if permuted), $\mu_{c}^{r_{2}}=\mu_{c}^{r_{4}}=0$ eV, $\mu_{c0}=0.4$ eV	239${}^{\circ }$	$\sigma_{x}$	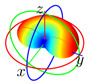	
2	17.3	1.51	−10.8	49.3	$\mu_{c}^{r_{4}}=0.4$ eV, $\mu_{c}^{r_{2}}=0$ eV, (flipped lobe if permuted), $\mu_{c}^{r_{1}}=\mu_{c}^{r_{3}}=0$ eV, $\mu_{c0}=1.2$ eV	183${}^{\circ }$	$\sigma_{y}$	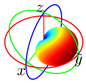	
3	1	1.63	−10.4	51.6	$\mu_{c}^{r_{1}}=\mu_{c}^{r_{3}}=0.6$ eV, $\mu_{c}^{r_{2}}=\mu_{c}^{r_{4}}=0$ eV, $\mu_{c0}=1.2$ eV	79${}^{\circ }$	$C_{2z}$, $\sigma_{x}$, $\sigma_{y}$	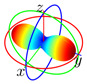	
4	1	0.86	−7.85	29.1	$\mu_{c}^{r_{2}}=\mu_{c}^{r_{4}}=0.4$ eV, $\mu_{c}^{r_{1}}=\mu_{c}^{r_{3}}=0$ eV, $\mu_{c0}=0.8$ eV	73${}^{\circ }$	$C_{2z}$, $\sigma_{x}$, $\sigma_{y}$	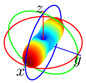	
5	17.8	1.42	−7.83	27.9	$\mu_{c}^{r_{1}}=\mu_{c}^{r_{3}}=1.2$ eV, $\mu_{c}^{r_{4}}=0.5$ eV, $\mu_{c}^{r_{2}}=0$ eV, $\mu_{c0}=1.2$ eV	120${}^{\circ }$	$\sigma_{y}$	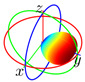	
BSR ($\phi =45^{\circ }$)	10.1	1.22	−10.7	39.5	$\mu_{c}^{r_{1}}=1.2$ eV, $\mu_{c}^{r_{4}}=0.3$ eV, $\mu_{c}^{r_{2}}=\mu_{c}^{r_{3}}=0$ eV, $\mu_{c0}=0.4$ eV	134${}^{\circ }$		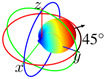	

**Table 9:** Chemical potentials of graphene parts for setting maximum gain angle $\phi_{\max}$ to $0^{\circ }$, $45^{\circ }$, and $90^{\circ }$.

$\phi_{\max}$	Chemical Potentials
0${}^{\circ }$	$\mu_{c}^{r_{1}}=0$ eV, $\mu_{c}^{r_{3}}=1.2$ eV, $\mu_{c}^{r_{2}}=\mu_{c}^{r_{4}}=0$ eV, and $\mu_{c0}=0.4$ eV
45${}^{\circ }$	$\mu_{c}^{r_{1}}=0$ eV, $\mu_{c}^{r_{3}}=1.2$ eV, $\mu_{c}^{r_{2}}=0$ eV, $\mu_{c}^{r_{4}}=0.3$ eV, and $\mu_{c0}=0.4$ eV
90${}^{\circ }$	$\mu_{c}^{r_{1}}=\mu_{c}^{r_{3}}=0$ eV, $\mu_{c}^{r_{2}}=0$ eV, $\mu_{c}^{r_{4}}=0.4$ eV, and $\mu_{c0}=1.2$ eV

## Data Availability

Data is contained within the article.

## References

[1] Wang Y., Chang B., Xue J., Cao X., Xu H., He H., Cui W., He Z. (2022). Sensing and slow light applications based on graphene metasurface in terahertz. Diam. Relat. Mater..

[2] Auton G., But D.B., Zhang J., Hill E., Coquillat D., Consejo C., Nouvel P., Knap W., Varani L., Teppe F. (2017). Terahertz Detection and Imaging Using Graphene Ballistic Rectifiers. Nano Lett..

[3] Prabhu S.S., Gupta V. (2018). Chapter 4—Terahertz Spectroscopy: Advances and Applications. Molecular and Laser Spectroscopy.

[4] Ullah Z., Witjaksono G., Nawi I., Tansu N., Irfan Khattak M., Junaid M. (2020). A Review on the Development of Tunable Graphene Nanoantennas for Terahertz Optoelectronic and Plasmonic Applications. Sensors.

[5] Dmitriev V., Rodrigues N.R.N.M., de Oliveira R.M.S., Paiva R.R. (2020). Graphene Rectangular Loop Antenna for Terahertz Communications. IEEE Trans. Antennas Propag..

[6] Shihzad W., Ullah S., Ahmad A., Abbasi N.A., Choi D.y. (2022). Design and Analysis of Dual-Band High-Gain THz Antenna Array for THz Space Applications. Appl. Sci..

[7] Chemweno E., Kumar P., Afullo T. (2023). Design of high-gain wideband substrate integrated waveguide dielectric resonator antenna for D-band applications. Optik.

[8] Grigorenko A., Polini M., Novoselov K. (2012). Graphene plasmonics. Nat. Photonics.

[9] Gonçalves P.A.D., Peres N.M.R. (2016). An Introduction to Graphene Plasmonics.

[10] Dash S., Patnaik A., Kaushik B.K. (2019). Performance enhancement of graphene plasmonic nanoantennas for THz communication. IET Microwaves Antennas Propag..

[11] Rudrapati R., Ameen S., Akhtar M.S., Shin H.S. (2020). Graphene: Fabrication Methods, Properties, and Applications in Modern Industries. Graphene Production and Application.

[12] Goyal R., Vishwakarma D.K. (2018). Design of a graphene-based patch antenna on glass substrate for high-speed terahertz communications. Microw. Opt. Technol. Lett..

[13] Ye R., James D.K., Tour J.M. (2019). Laser-induced graphene: From discovery to translation. Adv. Mater..

[14] Catania F., Marras E., Giorcelli M., Jagdale P., Lavagna L., Tagliaferro A., Bartoli M. (2021). A Review on Recent Advancements of Graphene and Graphene-Related Materials in Biological Applications. Appl. Sci..

[15] Balanis C.A. (2005). Antenna Theory: Analysis and Design.

[16] Novotny L., van Hulst N. (2011). Antennas for light. Nat. Photonics.

[17] Chang Y.-L., Chu Q.-X., Li Y. (2020). Reconfigurable dipole antenna array with shared parasitic elements for 360^∘^ beam steering. Int. J. Microw. Comput.-Aided Eng..

[18] Perruisseau-Carrier J. Graphene for antenna applications: Opportunities and challenges from microwaves to THz. Proceedings of the 2012 Loughborough Antennas & Propagation Conference (LAPC).

[19] Yang Y.-J., Wu B., Zhao Y.-T., Chi-Fan (2021). Dual-band beam steering THz antenna using active frequency selective surface based on graphene. EPJ Appl. Metamat..

[20] Wu Y., Qu M., Jiao L., Liu Y., Ghassemlooy Z. (2016). Graphene-based Yagi-Uda antenna with reconfigurable radiation patterns. AIP Adv..

[21] Dash S., Psomas C., Patnaik A., Krikidis I. (2022). An ultra-wideband orthogonal-beam directional graphene-based antenna for THz wireless systems. Sci. Rep..

[22] Rodrigues N.R.N.M., de Oliveira R.M.S., Dmitriev V. (2018). Smart Terahertz Graphene Antenna: Operation as an Omnidirectional Dipole and as a Reconfigurable Directive Antenna. IEEE Antennas Propag. Mag..

[23] Varshney G. (2020). Reconfigurable graphene antenna for THz applications: A mode conversion approach. Nanotechnology.

[24] Al-Shalaby N.A., Elhenawy A.S., Zainud-Deen S.H., Malhat H.A. (2021). Electronic Beam-Scanning Strip-Coded Graphene Leaky-Wave Antenna Using Single Structure. Plasmonics.

[25] Basiri R., Zareian-Jahromi E., Aghazade-Tehrani M. (2022). A reconfigurable beam sweeping patch antenna utilizing parasitic graphene elements for terahertz applications. Photonics Nanostruct.-Fundam. Appl..

[26] Shalini M., Madhan M.G. (2022). Photoconductive bowtie dipole antenna incorporating photonic crystal substrate for Terahertz radiation. Opt. Commun..

[27] Nissiyah G.J., Madhan M.G. (2019). A narrow spectrum terahertz emitter based on graphene photoconductive antenna. Plasmonics.

[28] Khaleel S.A., Hamad E.K., Parchin N.O., Saleh M.B. (2022). Programmable Beam-Steering Capabilities Based on Graphene Plasmonic THz MIMO Antenna via Reconfigurable Intelligent Surfaces (RIS) for IoT Applications. Electronics.

[29] Jiang Z., Wang Y., Chen L., Yu Y., Yuan S., Deng W., Wang R., Wang Z., Yan Q., Wu X. (2021). Antenna-integrated silicon–plasmonic graphene sub-terahertz emitter. APL Photonics.

[30] Chen P.Y., Alù A. (2013). A terahertz photomixer based on plasmonic nanoantennas coupled to a graphene emitter. Nanotechnology.

[31] Casiraghi C., Hartschuh A., Lidorikis E., Qian H., Harutyunyan H., Gokus T., Novoselov K.S., Ferrari A. (2007). Rayleigh imaging of graphene and graphene layers. Nano Lett..

[32] Hosseininejad S.E., Neshat M., Faraji-Dana R., Lemme M., Haring Bolivar P., Cabellos-Aparicio A., Alarcon E., Abadal S. (2018). Reconfigurable THz Plasmonic Antenna Based on Few-Layer Graphene with High Radiation Efficiency. Nanomaterials.

[33] Barybin A.A., Dmitriev V.A. (2002). Modern Electrodynamics and Coupled-Mode Theory: Application to Guided-Wave Optics.

[34] Overvig A.C., Malek S.C., Carter M.J., Shrestha S., Yu N. (2020). Selection rules for quasibound states in the continuum. Phys. Rev. B.

[35] Martí I.L., Kremers C., Cabellos-Aparicio A., Jornet J.M., Alarcón E., Chigrin D.N. Scattering of terahertz radiation on a graphene-based nano-antenna. Proceedings of the AIP Conference Proceedings. American Institute of Physics.

[36] Cao Y.S., Jiang L.J., Ruehli A.E. (2016). An equivalent circuit model for graphene-based terahertz antenna using the PEEC method. IEEE Trans. Antennas Propag..

[37] Llatser I., Kremers C., Cabellos-Aparicio A., Jornet J.M., Alarcón E., Chigrin D.N. (2012). Graphene-based nano-patch antenna for terahertz radiation. Photonics Nanostruct.-Fundam. Appl..

[38] Llatser I., Kremers C., Chigrin D.N., Jornet J.M., Lemme M.C., Cabellos-Aparicio A., Alarcon E. (2012). Radiation characteristics of tunable graphennas in the terahertz band. Radioengineering.

[39] Zhang B., Zhang J., Liu C., Wu Z.P., He D. (2018). Equivalent resonant circuit modeling of a graphene-based bowtie antenna. Electronics.

[40] Rakheja S., Sengupta P., Shakiah S.M. (2020). Design and Circuit Modeling of Graphene Plasmonic Nanoantennas. IEEE Access.

[41] Garcia M.E.C., de Oliveira R.M.S., Rodrigues N.R.N.M. (2022). Semi-analytical Equations for Designing Terahertz Graphene Dipole Antennas on Glass Substrate. J. Microwaves, Optoelectron. Electromagn. Appl..

[42] Christensen J., Manjavacas A., Thongrattanasiri S., Koppens F.H.L., de Abajo F.J.G. (2012). Graphene Plasmon Waveguiding and Hybridization in Individual and Paired Nanoribbons. ACS Nano.

[43] Correas-Serrano D., Gomez-Diaz J.S., Perruisseau-Carrier J., Alvarez-Melcon A. (2014). Graphene-based plasmonic tunable low-pass filters in the terahertz band. IEEE Trans. Nanotechnol..

[44] Powell M.J.D. (1981). Approximation Theory and Methods.

[45] de Oliveira R.M.S., Rodrigues N.R.N.M., Dmitriev V. (2015). FDTD Formulation for Graphene Modeling Based on Piecewise Linear Recursive Convolution and Thin Material Sheets Techniques. IEEE Antennas Wirel. Propag. Lett..

[46] Taflove A., Hagness S.C. (2005). Computational Electrodynamics, The Finite-Difference Time-Domain Method.

[47] Gevorgian S., Berg H. Line Capacitance and Impedance of Coplanar-Strip Waveguides on Substrates with Multiple Dielectric Layers. Proceedings of the 2001 31st European Microwave Conference.

[48] Binns K.J., Lawrenson P.J. Analysis and Computation of Electric and Magnetic Field Problems; Pergamon International Library of Science, Technology, Engineering and Social Studies: 2013. https://www.sciencedirect.com/book/9780080166384/analysis-and-computation-of-electric-and-magnetic-field-problems.

[49] Gradshteyn I.S., Ryzhik I.M. (2000). Tables of Integrals, Series, and Products.

[50] Whitaker J.C. (2005). The Electronics Handbook.

[51] Dmitriev V., Castro W., Melo G., Oliveira C. (2020). Controllable graphene W-shaped three-port THz circulator. Photonics Nanostruct.-Fundam. Appl..

[52] Huang J., Song Z. (2021). Terahertz graphene modulator based on hybrid plasmonic waveguide. Phys. Scr..

[53] Hou H., Teng J., Palacios T., Chua S. (2016). Edge plasmons and cut-off behavior of graphene nano-ribbon waveguides. Opt. Commun..

[54] Hong J.S., Lancaster M.J. (2001). Microstrip Filters for RF/Microwave Applications.

[55] Li D., Huang Y., Shen Y.C., Khiabani N. Effects of substrate on the performance of photoconductive THz antennas. Proceedings of the 2010 International Workshop on Antenna Technology.

[56] Adekoya G.J., Sadiku R.E., Ray S.S. (2021). Nanocomposites of PEDOT:PSS with Graphene and its Derivatives for Flexible Electronic Applications: A Review. Macromol. Mater. Eng..

[57] Gomez-Diaz J., Moldovan C., Capdevila S., Romeu J., Bernard L., Magrez A., Ionescu A., Perruisseau-Carrier J. (2015). Self-biased reconfigurable graphene stacks for terahertz plasmonics. Nat. Commun..

[58] Li Y., Yu H., Dai T., Jiang J., Wang G., Yang L., Wang W., Yang J., Jiang X. (2016). Graphene-Based Floating-Gate Nonvolatile Optical Switch. IEEE Photonics Technol. Lett..

[59] Fuscaldo W., Burghignoli P., Baccarelli P., Galli A. (2017). Graphene Fabry-Perot Cavity Leaky-Wave Antennas: Plasmonic Versus Nonplasmonic Solutions. IEEE Trans. Antennas Propag..

[60] McPherson J.W., Kim J., Shanware A., Mogul H., Rodriguez J. (2003). Trends in the ultimate breakdown strength of high dielectric-constant materials. IEEE Trans. Electron Devices.

[61] Kong W., Kum H., Bae S.H., Shim J., Kim H., Kong L., Meng Y., Wang K., Kim C., Kim J. (2019). Path towards graphene commercialization from lab to market. Nat. Nanotechnol..

[62] Meng Y., Feng J., Han S., Xu Z., Mao W., Zhang T., Kim J.S., Roh I., Zhao Y., Kim D.H. (2023). Photonic van der Waals integration from 2D materials to 3D nanomembranes. Nat. Rev. Mater..

